# City-level meteorological conditions modify the relationships between exposure to multiple air pollutants and the risk of pediatric hand, foot, and mouth disease in the Sichuan Basin, China

**DOI:** 10.3389/fpubh.2023.1140639

**Published:** 2023-07-11

**Authors:** Wennian Cai, Caiying Luo, Xiaoran Geng, Yuanyi Zha, Tao Zhang, Huadong Zhang, Changhong Yang, Fei Yin, Yue Ma, Tiejun Shui

**Affiliations:** ^1^West China School of Public Health and West China Fourth Hospital, Sichuan University, Chengdu, China; ^2^Graduate School of Kunming Medical University, Kunming, China; ^3^Chongqing Center for Disease Control and Prevention, Chongqing, China; ^4^Sichuan Center for Disease Control and Prevention, Chengdu, China; ^5^Yunnan Center for Disease Control and Prevention, Kunming, China

**Keywords:** meteorological conditions, air pollution-HFMD associations, modification effects, air pollution, two-stage analysis

## Abstract

**Background:**

Several studies have examined the effects of city-level meteorological conditions on the associations between meteorological factors and hand, foot, and mouth disease (HFMD) risk. However, evidence that city-level meteorological conditions modify air pollutant-HFMD associations is lacking.

**Methods:**

For each of the 17 cities in the Sichuan Basin, we obtained estimates of the relationship between exposures to multiple air pollutants and childhood HFMD risk by using a unified distributed lag nonlinear model (DLNM). Multivariate meta-regression models were used to identify the effects of city-level meteorological conditions as effect modifiers. Finally, we conducted subgroup analyses of age and sex to explore whether the modification effects varied in different subgroups.

**Results:**

The associations between PM_2.5_/CO/O_3_ and HFMD risk showed moderate or substantial heterogeneity among cities ( $I^{2}$ statistics: 48.5%, 53.1%, and 61.1%). Temperature conditions significantly modified the PM_2.5_-HFMD association, while relative humidity and rainfall modified the O_3_-HFMD association. Low temperatures enhanced the protective effect of PM_2.5_ exposure against HFMD risk [PM_2.5_ <32.7  μg/m^3^ or PM_2.5_ >100  μg/m^3^, at the 99th percentile: relative risk (RR) = 0.14, 95% CI: 0.03–0.60]. Low relative humidity increased the adverse effect of O_3_ exposure on HFMD risk (O_3_ >128.7 μg/m^3^, at the 99th percentile: RR = 2.58, 95% CI: 1.48–4.50). However, high rainfall decreased the risk of HFMD due to O_3_ exposure (O_3_: 14.1–41.4  μg/m^3^). In addition, the modification effects of temperature and relative humidity differed in the female and 3–5  years-old subgroups.

**Conclusion:**

Our findings revealed moderate or substantial heterogeneity in multiple air pollutant-HFMD relationships. Temperature, relative humidity, and rainfall modified the relationships between PM_2.5_ or O_3_ exposure and HFMD risk.

## 1. Introduction

Hand, foot, and mouth disease (HFMD), a widespread viral infectious disease, mainly affects children through enteroviruses (1–3), such as enterovirus 71 and coxsackievirus A16. HFMD may be transmitted via the fecal-oral route or respiratory droplets. Over the past few decades, many countries and regions, especially the Asia-Pacific region (4, 5), have experienced large HFMD outbreaks. Since 2008, HFMD has been listed as a notifiable Class C infectious disease in China. In 2019, the number of HFMD cases reached 1,918,830, and the incidence rate was 137.4 per 100,000 people, ranking second among all notifiable infectious diseases in China (6). The high incidence rate and lack of specific drug treatments indicate that HFMD still imposes a substantial public health burden in China.

Many studies have explored the environmental risk factors for HFMD. Meteorological factors, especially temperature and relative humidity, have been widely studied (7–12). With rapid increases in industrialization and urbanization, ambient air pollution has been exacerbated. Growing evidence indicates that air pollution may cause adverse health outcomes (13–15). For instance, Ibrahim et al. (16) found that exposure to fine particulate matter (diameter ≤2.5 μm; PM_2.5_) and SO_2_ increased the impacts of respiratory diseases in hospitalized children. Children under 5 years old are vulnerable to HFMD. As their lungs may still be developing and their immune defenses are immature, they are more likely to be adversely affected by exposure to air pollution (17–19). Therefore, the short-term exposure-response associations between air pollutants and pediatric HFMD risk has received increasing attention in recent years (20–22).

However, previous studies have indicated that the exposure-response relationships between air pollutants and HFMD had heterogeneity. For instance, Huang et al. (23) found that PM_10_ was not significantly related to HFMD morbidity. However, Peng et al. (20) found that extremely high or low concentrations of PM_10_ had adverse effects on HFMD. Furthermore, a seasonal analysis revealed that the effect of PM_10_ exposure on the relative risk (RR) of HFMD was significantly higher in the cold season (24). Regarding the O_3_-HFMD association, Gu et al. (25) found that O_3_ exposure was significantly associated with HFMD (RR = 2.12, 95% CI: 1.47–3.05), while Yan et al. (21) found that O_3_ exposure (>104 μg/m^3^) reduced HFMD risk. The heterogeneity of short-term associations may be due to the modification effects of meteorological conditions (12, 26–29). Previous studies have suggested that meteorological conditions may affect the relationship between air pollutant exposure and respiratory health. A study found that the relationship between chronic bronchitis and total suspended particulate matter (TSP) may be modified by humidity. Lower humidity may exacerbate the hazardous effects of TSP (30). Another study found that temperature may modify the effect of the air pollution index (API) on respiratory morbidity (31). Therefore, we predicted that meteorological conditions may also modify the relationships between air pollutant exposure and HFMD risk. Regarding the meteorological factor-HFMD associations, a previous study suggested that the diurnal temperature range modified the relationship between temperature and pediatric HFMD (32). Another study found that city-specific climate indicators, including temperature, sunshine duration, and air pressure, modified the relative humidity-HFMD association (27). However, to our knowledge, there is no evidence that meteorological conditions modify the air pollutant-HFMD associations.

The Sichuan Basin, a region with the fourth-highest air pollution in China, contains approximately 90% of the resident population of Sichuan Province (33). Located in the subtropical monsoon region, with deep mountain-basin topographic characteristics, the Sichuan Basin has a low average wind speed and a foggy and wet climate. These complex topographic and meteorological conditions make air stagnation more likely and prevent diffusion of air pollutants in the basin after excessive emission (33–36).

The objective of this study was to examine the modification effects of meteorological conditions as effect modifiers on air pollutant-HFMD associations. We adopted a multicity, unified distributed lag nonlinear model (DLNM)-based, two-stage time series study of 17 cities in the Sichuan Basin. In addition, since it is well known that meteorological factors are important environmental factors associated with HFMD, we simultaneously considered these meteorological factors to provide a more comprehensive understanding of HFMD risk in the Sichuan Basin.

## 2. Methods

### 2.1. Study area

The Sichuan Basin is in eastern Sichuan Province, which is located in southwestern China, with an area of approximately 160,000 square kilometers. We analyzed 17 prefecture-level cities in the Sichuan Basin, which is in the subtropical monsoon zone and has a humid climate. The meteorological conditions of cities in the Sichuan Basin were based on the latitudinal zonal climate, as the layered zonal climate varied (33, 37).

### 2.2. Data collection

Daily HFMD case counts of 17 cities from January 2015 to December 2017 were provided by the Sichuan Provincial Center for Disease Control and Prevention.^1^ This study analyzed HFMD cases in children aged 0–5 years. In the subgroup analysis of age, the cases were divided into three subgroups, age < 1 year, 1 ≤ age < 3 years, and 3 ≤ age < 6 years, to represent cases in infants, preschool children, and kindergarten children, respectively.

Daily meteorological data were extracted from the National Meteorological Science Data Center.^2^ The variables included daily average temperature (°C), daily average relative humidity (%), sunshine duration (hours), daily average wind speed (m/s), daily average air pressure (hPa), and daily average rainfall (mm). Daily air pollutant data were collected from the Sichuan Environmental Monitoring Center,^3^ including the daily average SO_2_ concentration (μg/m^3^), daily average NO_2_ (μg/m^3^), daily average PM_10_ (μg/m^3^), daily average PM_2.5_ (μg/m^3^), daily average CO (mg/m^3^), daily average O_3_ (μg/m^3^), and daily air quality index (AQI). A detailed description of the air quality station and meteorological monitoring station in the Sichuan Basin is provided in Supplementary Tables S1, S2. For the city-level meteorological conditions, we calculated the arithmetic average of the daily data of the meteorological variables (26, 27, 32) (including temperature, relative humidity, sunshine duration, wind speed, and rainfall) of each city in the study period (Supplementary Table S3).

### 2.3. Statistical analysis

#### 2.3.1. Construction of a unified DLNM to obtain city-specific estimates

We used a DLNM in this study due to the nonlinearity and lag of HFMD surveillance data. Proposed by Gasparrini et al. (38–40), DLNMs can simultaneously consider nonlinear associations and lagged effects between environmental exposure and health outcomes. Currently, DLNMs are commonly used for environmental and epidemiological time series analysis (28, 41).

Spearman correlation analysis showed that PM_10_ concentrations, PM_2.5_ concentrations, and AQI values were highly correlated. Thus, PM_2.5_ concentrations were selected for further analysis. The city-specific time series HFMD case data were overdispersed (Supplementary Table S4), so we adopted a common DLNM based on a quasi-Poisson distribution (38, 42) to investigate the exposure-lag response relationships between meteorological factors, air pollutants, and HFMD case counts for each city. Referring to prior knowledge (8, 26) and the results of a systematic sensitivity analysis (Supplementary Text S1, Supplementary Figures S1–S7, and Supplementary Tables S5–S8), we determined the DLNM, which was constructed as follows:


$$Y_{t}\sim Quasi-poisson\left(\mu_{t}\right)$$

$$\begin{array}{l} \log\left(\mu_{t}\right)=\alpha +\sum cb\left(M_{t},lag\right)+Confounder_{t}+Auto_{t}\\ \;\;\;\;\;\;\;\;\;\;\;\;\;\;\;\;\;\;\;+ns\;\left(time,df\right)+Dow_{t}+Holiday_{t} \end{array}$$

where $Y_{t}$ represents the HFMD case count on day t ; α is the intercept term; and $M_{t}$ is the examined variables of meteorological factors and air pollution, including temperature, relative humidity, wind speed, and concentrations of PM_2.5_, CO and O_3_. cb(.) is the cross-basis function, which can simultaneously describe the relationship between the exposure-response dimension and the lag-response dimension between the explanatory variable and the dependent variable. The exposure dimension used the natural cubic spline (*ns*) function with two equal knots. The lag dimension also adopted the *ns* function with 3 degrees of freedom ( df ). The lag period was defined as 0 to 14 days, which encompasses the incubation and infectious periods of HFMD (20, 22). The average incubation period of HFMD is usually defined as 3–5 days (43), and the disease usually ends within 7 to 10 days (44). ${\mathrm{Confounder}}_{t}$ contains the sunshine duration, rainfall, and concentrations of NO_2_ and SO_2_. Sunshine duration, NO_2_ and SO_2_ were controlled by calculating a simple moving average with a lag period of 0 to 14 days. Rainfall was determined by calculating the exponential moving average with a lag period of 0 to 14 days and adopting the *ns* function with 3 df . The seasonality and long-term trend used an *ns* function with 8 df to control annually. ${\mathrm{Auto}}_{t}$ is an autoregressive term that converted the daily cases data to a logarithmic scale and incorporated the first and second lags; ${\mathrm{Dow}}_{t}$ indicates the day of the week, and ${\mathrm{Holiday}}_{t}$ is the national holiday date.

#### 2.3.2. Pooled city-specific estimates and exploration of the modification effects of city-level meteorological conditions by multivariate meta-regression models

We summed the lagged effects over 0–14 days to obtain the cumulative associations for each city. The overall cumulative exposure-response associations of the Sichuan Basin were obtained by pooling the city-specific estimates (39, 40). The Cochran *Q* test was performed to examine whether the heterogeneity of city-specific cumulative associations was statistically significant, and $I^{2}$ statistics were calculated to determine the proportion of heterogeneity in the total variation (45, 46). In this study, we determined the grade of heterogeneity by Cochrane’s evaluation criteria (47). Taking the intercept-only meta-regression model as a reference, multivariate meta-regression models were further conducted to analyze the modification effect of meteorological conditions. As meta-predictors, meteorological conditions were added to fit the meta-regression model individually. We selected the likelihood ratio (LR) test to evaluate the modification effect. Meteorological condition variables with *p* < 0.2 in the LR test were included in the subsequent multiple meta-predictor regression model as alternative variables. We fitted all candidate subsets in the models, and the best multiple meta-regression model was identified as the one with the lowest Akaike information criterion (AIC). AIC values reflect the goodness of fit of models. Moreover, Δ*I*^2^ was calculated to determine the proportion of the heterogeneity explained by the modifier. All of the statistical analyses in this study were performed in R software (4.0.3) using the *dlnm* and *mvmeta* packages, and statistical significance was tested as *p* < 0.05.

## 3. Results

### 3.1. Descriptive statistics of daily HFMD case counts

**Figure 1:**
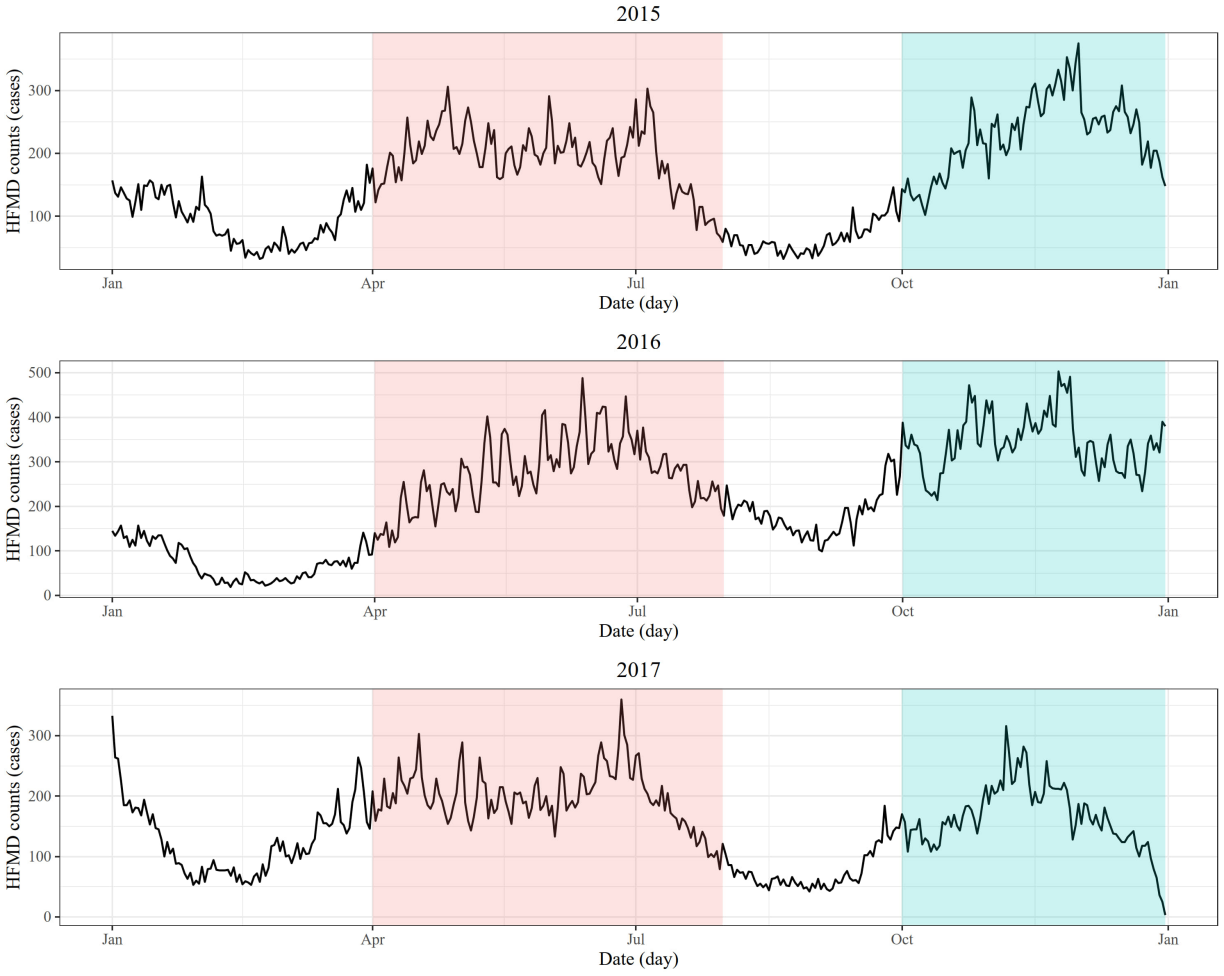
Daily time series of the number of HFMD cases in 2015, 2016, and 2017 (red shade: April-July, blue shade: October-December).

From January 1st, 2015 to December 31st, 2017, the total number of HFMD cases in 17 prefecture-level cities in the Sichuan Basin was 187,823. The time series distribution of HFMD case counts showed an effect of season. There were two peaks in HFMD case counts throughout the year. One peak was in April–July (summer), and the other was October–December (winter) (Figure 1).

### 3.2. The heterogeneity of overall pooled estimates of the exposure-response relationships

Table 1 shows the heterogeneity results of the associations between the overall cumulative meteorological factors and air pollutants with HFMD risk. The exposure-response associations of all study variables had statistically significant heterogeneity. The proportions of heterogeneity (in terms of *I^2^* values) in the temperature-HFMD, wind speed-HFMD, and relative humidity-HFMD associations were 31.3%, 34.9%, and 54.1%, respectively. The association between PM_2.5_ concentrations and HFMD risk showed moderate heterogeneity, with an $I^{2}$ value of 48.5%. The associations of CO and O_3_ concentrations with HFMD risk showed substantial heterogeneity, with $I^{2}$ values of 53.1% and 61.1%, respectively.

**Table 1:** The heterogeneity results of the overall cumulative exposure-response associations.

Variable	Cochran *Q* test			Model fit	Heterogeneity	Heterogeneity grade
	*Q*	*df*	*p*	AIC	*I*^2^(%)	(Cochrane’s evaluation criteria)
Temperature	69.85	48	0.021	134.89	31.3	Moderate
Wind speed	73.73	48	0.010	149.23	34.9	Moderate
Relative humidity	104.59	48	<0.001	149.92	54.1	Substantial
PM_2.5_	93.24	48	<0.001	156.60	48.5	Moderate
CO	102.27	48	<0.001	157.84	53.1	Substantial
O_3_	123.35	48	<0.001	150.48	61.1	Substantial

### 3.3. Modification effects of meteorological conditions on the overall cumulative relationships among air pollutants, meteorological factors and HFMD

Table 2 shows significant modification effects of the meteorological conditions on air pollutant-HFMD associations. The ΔAICs of all models indicated that after adding the meta-predictors, the model fit improved. Temperature (*p* = 0.037) modified the PM_2.5_-HFMD association, with explaining 1% of the heterogeneity ( $I^{2}$ statistic). Relative humidity (*p* = 0.047) and rainfall (*p* = 0.028) individually modified the O_3_-HFMD association, and the Δ*I*^2^ values showed proportions of explained heterogeneity of 2.6% and 3.5%. The joint modification effects of relative humidity, wind speed, and rainfall on the O_3_-HFMD association explained more of the heterogeneity than any single effect modifier, explaining 11.2% of the heterogeneity.

**Table 2:** Significant meteorological condition modifiers and multivariate meta-regression model results.

Exposure	Modifier	LR test			Cochran *Q* test			Model fit		Heterogeneity	
		*Stats*	*df*	*p*	*Q*	*df*	*p*	AIC	ΔAIC	*I*^2^	Δ*I*^2^
Single meta-predictor model											
Temperature	Reference				69.85	48	0.021	134.89	—	31.3	—
	Temperature	8.64	3	0.034	58.89	45	0.080	132.25	−2.64	23.6	−7.7
Wind speed	Reference	—	—	—	73.73	48	0.010	149.23	—	34.9	—
	Relative humidity	9.19	3	0.027	53.94	45	0.170	146.04	−3.19	16.6	−18.3
PM_2.5_	Reference	—	—	—	93.24	48	<0.001	156.60	—	48.5	—
	Temperature	8.49	3	0.037	85.79	45	<0.001	154.11	−2.49	47.5	−1
O_3_	Reference	—	—	—	123.35	48	<0.001	150.48	—	61.1	—
	Relative humidity	7.97	3	0.047	108.55	45	<0.001	148.52	−1.96	58.5	−2.6
	Rainfall	9.09	3	0.028	106.09	45	<0.001	147.39	−3.09	57.6	−3.5
Multiple meta-predictors model											
Temperature	Temperature + relative humidity	14.85	6	0.021	51.02	42	0.16	132.03	−2.86	17.7	−13.6
O_3_	Relative humidity + wind speed + rainfall	23.26	9	0.006	77.79	39	<0.001	145.23	−5.25	49.9	−11.2

In addition, meteorological conditions modified the meteorological factor-HFMD relationships. Temperature-HFMD and wind speed-HFMD associations were modified by temperature (*p* = 0.034) and relative humidity (*p* = 0.027). The results of Δ*I*^2^ showed proportions of explained heterogeneity of 7.7 and 18.3%, respectively. The joint modification effects of temperature and relative humidity on the temperature-HFMD association showed that 13.6% of heterogeneity was explained. Complete results of the modification effects of meteorological conditions on the meteorological factor/air pollutant-HFMD associations are described in Supplementary Tables S9–S14.

**Figure 2:**
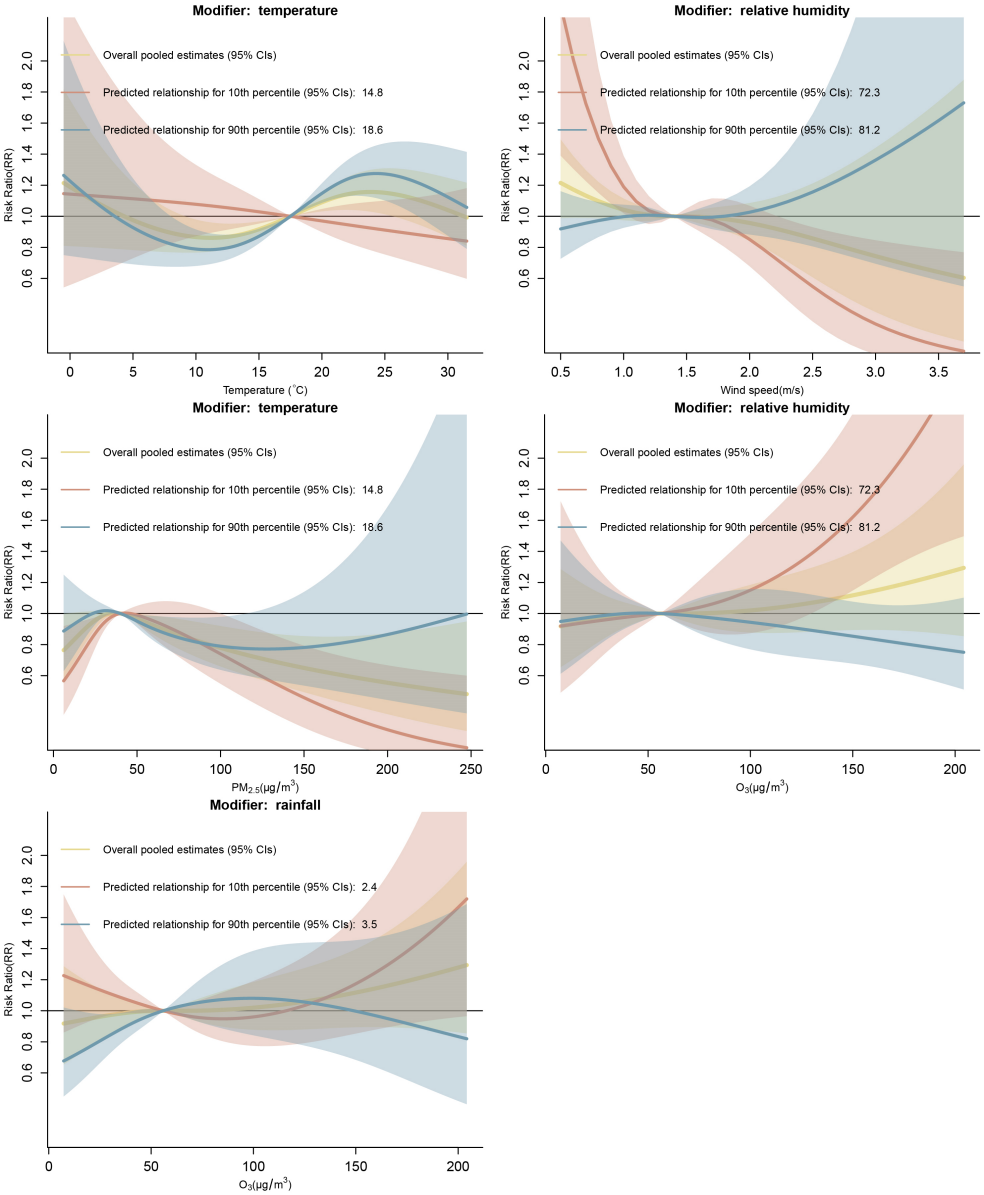
At the 10th and 90th percentiles, meteorological conditions modified the pooled cumulative exposure-HFMD associations (median value of the variables set as reference).

Using the pooled cumulative exposure-HFMD relationship curves as a reference, predicted relationship curves at the 10th and 90th percentiles of statistically significant meteorological conditions were drawn separately, as shown in Figure 2. Temperature, relative humidity, and rainfall modified the relationship between air pollutant exposures and HFMD risk. Temperature modified the PM_2.5_-HFMD association. Predicted at the 10th percentile (14.8°C), temperature significantly enhanced the protective effects of PM_2.5_ exposure against HFMD risk under high and low levels of PM_2.5_ concentrations (PM_2.5_ <32.7 μg/m^3^ or PM_2.5_ >100 μg/m^3^). At the 99th percentile of PM_2.5_ concentrations, the RR was 0.14 (95% CI: 0.03–0.60). The plot showed that the RR was decreasing, and the predicted curve became steeper. At the 90th percentile (18.6°C) of temperature, we found only a very small range of PM_2.5_ concentrations in which the predicted curve was statistically significant. For the modification effects of relative humidity on the O_3_-HFMD association, at the 10th percentile, relative humidity showed an enhanced effect on the right side, with the RR increasing quickly when the O_3_ concentration was higher than 129.7 μ g /m^3^. At the 99th percentile of O_3_ concentrations, the RR was 2.58 (95% CI: 1.48–4.50). Rainfall also modified the O_3_-HFMD association. At the 90th percentile, rainfall significantly modified the O_3_-HFMD association at low O_3_ concentrations (14.1–41.4 μg/m^3^), enhancing the protective effect of O_3_. At the 5th percentile of O_3_ concentrations, the RR was 0.75 (95% CI: 0.56–0.99).

**Figure 3:**
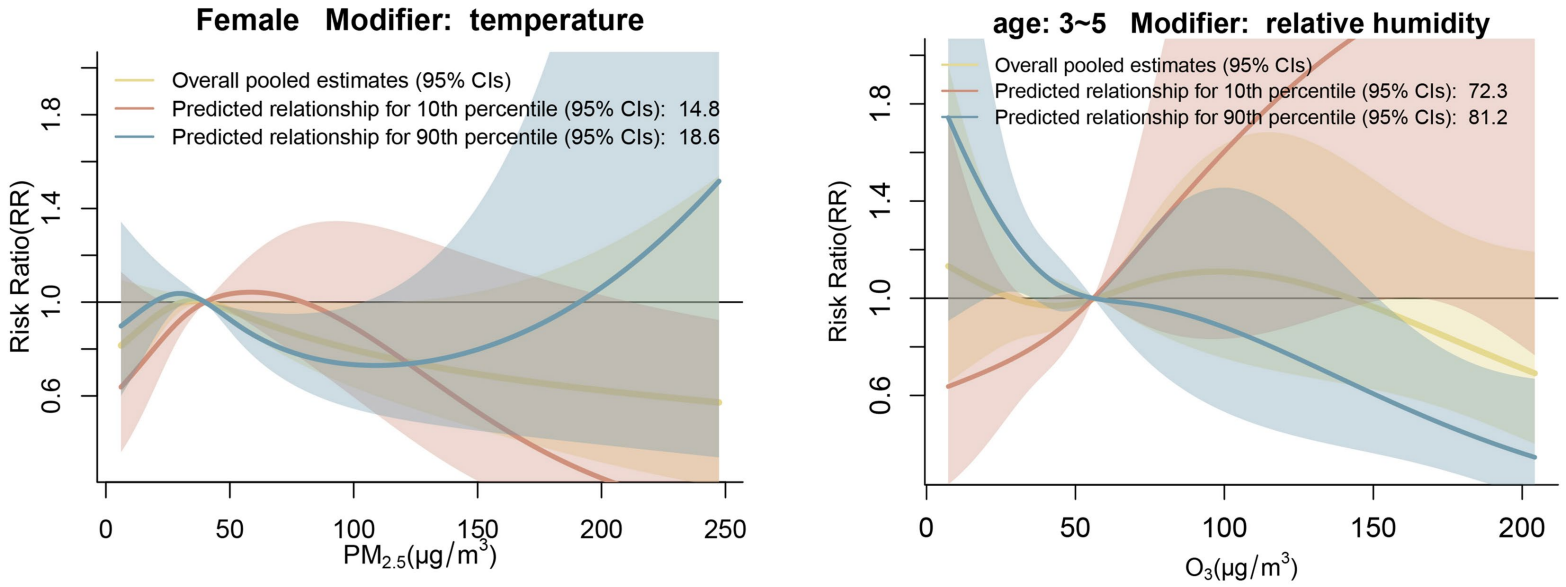
Subgroup analysis by gender and age At the 10th and 90th percentiles, meteorological conditions modified the pooled cumulative exposure-HFMD associations.

Figure 3 shows the results of subgroup analyses. We observed that temperature modified the PM_2.5_-HFMD association in the female subgroup. In the age subgroup analysis, we observed that relative humidity modified the O_3_-HFMD association in the 3–5 years-old subgroup at the 90th percentile, and a protective effect was found at high O_3_ concentrations, in contrast to other age subgroup analyses.

For the meteorological factor-HFMD associations, temperature and relative humidity were effect modifiers. At the 90th percentile (18.6°C), temperature significantly modified the temperature-HFMD association, with greater modification effects at the high-temperature level. The effect mainly changed the magnitude of the RR rather than the shape of the predicted curve. At the 10th percentile (72.3%), relative humidity significantly modified the wind speed-HFMD association. At a relatively high wind speed (>2.3 m/s), the association curve became steeper, and the curve slope decreased, which indicated a strong protective effect of wind speed against HFMD risk. However, at the left tail of the predicted curve, relative humidity increased the adverse effect of wind speed on HFMD risk. We observed that when the wind speed was 0.5 m/s, the RR was 2.36 (95% CI: 1.39–4.00).

## 4. Discussion

We began by exploring the heterogeneity of short-term associations between exposure to different air pollutants, including PM_2.5_, CO, and O_3_, and the number of HFMD cases in the Sichuan Basin. We found that these associations had moderate or substantial heterogeneity, with $I^{2}$ statistics of 48.5%, 53.1%, and 61.1% for PM_2.5_, CO, and O_3_, respectively. Second, we analyzed the modification effects of meteorological conditions on the air pollutant-HFMD associations. Overall, temperature significantly modified the PM_2.5_-HFMD association, while relative humidity and rainfall modified the O_3_-HFMD association. To our knowledge, this is the first study to explore the modification effects of meteorological conditions on air pollutant-HFMD associations.

There is some evidence of heterogeneity in the meteorological factor-HFMD associations (9, 27, 48, 49). For example, a study in mainland China found that 77.8% of the residual heterogeneity in the relative humidity-HFMD association was attributable to differences between cities (27). However, studies on the heterogeneity of multiple air pollutant-HFMD associations are limited. Our multicity study showed that the effects of PM_2.5_, CO, and O_3_ concentrations on HFMD risk exhibited moderate or substantial heterogeneity, which may provide a reference for future research.

For the PM_2.5_-HFMD associations, we found that the association curve exhibited an inverse “V” shape and that low and high PM_2.5_ concentrations exerted a protective effect against HFMD risk. For the modification effects, we concluded that low temperature modified and strengthened the PM_2.5_-HFMD association. In contrast, a study in northeast Asia found that extremely high temperatures may exacerbate the adverse effects of air pollutants on human health (50). Other studies have found that temperature modified the effect of particulate matter on influenza incidence and cardiorespiratory disease mortality (51–53), but the underlying mechanisms remain unclear. Similarly, evidence that temperature modifies the PM_2.5_-HFMD association is complicated, and still not yet clear. However, there are some plausible explanations for our findings. It is well known that low temperatures may restrict the survival and reproduction of viruses (9, 12, 54). Therefore, during the cold period, the activity of viruses may have been inhibited by low temperatures. Additionally, people would have less opportunity to inhale particulate matter containing viruses, especially at low PM_2.5_ concentrations. Experimental evidence indicated that particulate matter led to airway hyperresponsiveness and oxidative stress (55, 56). However, during the cold period with heavy PM_2.5_ pollution, guardians of children would pay attention to the forecasted air quality and keep their children at home. This behavior greatly reduced the risk of exposure to heavy PM_2.5_ pollution of vulnerable HFMD populations in a cold environment.

For the pooled O_3_-HFMD associations, we found substantial heterogeneity. We further analyzed the modification effect of meteorological conditions and found that relative humidity and rainfall modified this association. Remarkably, low relative humidity intensified the adverse effect of high O_3_ concentrations on HFMD risk. Leitte et al. (30) also found that dry air may increase the risk of air pollutant exposure for respiratory health since the respiratory airways of individuals seem to be protected by higher humidity. Compared with fog–haze weather mainly caused by PM_2.5_, O_3_ pollution may not attract much attention from parents, and children may engage in more outdoor activity on days with low relative humidity, which may have a higher human body comfort index than that on days with high relative humidity. Hence, owing to the immature host defense system and easy infection by respiratory pathogens (57), susceptible children may have a higher risk of infection. Furthermore, Yin et al. (58) found that for each 10 μ g /m^3^ increase in O_3_ concentration, the estimated daily total mortality increased by 0.24%. Public health policymakers should devote more attention to specific meteorological conditions that aggravate the impact of high air pollutant concentrations on HFMD risk and develop targeted countermeasures. For example, during periods with severe ozone pollution and low relative humidity, programs to increase public awareness could be conducted in kindergartens, primary schools, etc. Children should be reminded to wear masks or reduce their outdoor activity in such conditions to reduce the health impacts of air pollution. From a sustainable development perspective, clean energy, such as nuclear and wind power, could be developed to reduce emissions from industries, vehicle exhaust, etc., and improve environmental quality (59). In this way, the influence of air pollution on HFMD risk may also be decreased.

In addition, we found that high rainfall conditions enhanced the protective effect of low O_3_ concentrations on HFMD risk. Heavy rainfall may restrict the outdoor activity of children, which may reduce exposure to O_3_ and further reduce the probability of contact between susceptible populations and infected children, thus decreasing the influence of O_3_ concentrations on HFMD risk. However, the exact mechanism by which rainfall conditions modify the O_3_-HFMD association is not clear and merits further study.

For the CO-HFMD association, although there was substantial heterogeneity among regions, we did not identify significant modification effects of meteorological conditions. The heterogeneity of the CO-HFMD association may be caused by other factors besides meteorological conditions, such as geographical factors.

This is the first study to explore the modification effect of city-level meteorological conditions on air pollutant-HFMD associations, expanding our understanding of the effect of air pollution on HFMD risk. Our findings have practical implications in two aspects. First, in the development of public health policies, our findings suggest that meteorological conditions and air pollutants jointly affected the incidence of HFMD. For instance, to mitigate the harmful effects of O_3_ concentrations on the incidence of HFMD, different policies should be implemented in the rainy and dry seasons. Second, in the development of individual-level interventions, our study can serve as a reference. Children should adopt healthy hygiene habits, such as washing their hands before meals and after going to the bathroom. Parents or guardians should take care to reduce children’s outdoor activities during HFMD outbreaks. In addition, it is essential to check the weather forecast and air quality before going outside.

However, some limitations should be acknowledged in our study. First, this is an ecological study. Due to the characteristics of our study design, our findings cannot be used to prove causality. Additionally, we did not consider spatial correlations in our study. This issue will be considered in further research.

## 5. Conclusion

Our findings suggest that meteorological conditions modified the air pollutant-HFMD associations in the Sichuan Basin. Specifically, temperature, relative humidity, and rainfall partly explained heterogeneity in the associations of PM_2.5_ or O_3_ concentrations with HFMD risk. The complex interaction between meteorological conditions and air pollution indicates that it is necessary to consider the joint effects of these environmental factors to obtain more accurate insights into HFMD risk. Our findings could inform the development of effective regional public health strategies for the prevention and control of HFMD.

## Data availability statement

The datasets presented in this article are not readily available because the datasets generated and/or analyzed in this study are available from Sichuan Center for Disease Control and Prevention. The authors used the data for this current study under a license from the Sichuan CDC, so the data cannot be shared publicly. Requests to access the datasets should be directed to https://www.sccdc.cn/.

## Ethics statement

Our study was carried out in accordance with relevant guidelines and regulations and approved by the Institutional Review Board of the School of Public Health, Sichuan University. The daily HFMD data used in this current study were aggregated at the city level by counts, so no confidential information was involved in this study, and the study was exempt from ethical approval procedures.

## Author contributions

WC, YM, and TS designed the study. CY, TZ, and HZ collected the data. WC, CL, and XG performed the analysis and interpreted the results. WC, YM, YZ, and FY coordinated and drafted the manuscript. All authors contributed to the article and approved the submitted version.

## Funding

This work was supported by the National Natural Science Foundation of China (Grant nos. 81872713 and 81803332), the Sichuan Science and Technology Program (Grant nos. 2021YFS0181 and 2022YFS0641), and Chongqing Science and Technology Program (Grant no. cstc2020jscx-cylhX0003; URL: http://kjj.cq.gov.cn/).

## Conflict of interest

The authors declare that the research was conducted in the absence of any commercial or financial relationships that could be construed as a potential conflict of interest.

## Publisher’s note

All claims expressed in this article are solely those of the authors and do not necessarily represent those of their affiliated organizations, or those of the publisher, the editors and the reviewers. Any product that may be evaluated in this article, or claim that may be made by its manufacturer, is not guaranteed or endorsed by the publisher.

## Footnotes

^1^ https://www.sccdc.cn/

^2^ http://data.cma.cn/

^3^ http://www.scaepp.cn/sthjt/c104334/scemc.shtml
