# Supplementary material for: City-level meteorological conditions modify the relationships between exposure to multiple air pollutants and the risk of pediatric hand, foot, and mouth disease in the Sichuan Basin, China
Source: Front Public Health. 2023 Jul 11;11:1140639. doi: 10.3389/fpubh.2023.1140639 (PMC10433208; doi:10.3389/fpubh.2023.1140639)
Supplement: Supplementary file 1 [file Data_Sheet_1.pdf]

Supplementary Material

The modification effects of city-level meteorological conditions on the relationships between multiple air pollutants and pediatric hand, foot, and mouth disease in the Sichuan Basin, China

Wennian Cai[1,†], Caiying Luo[1,†], Xiaoran Geng[1], Yuanyi Zha[2], Tao Zhang[1], Huadong Zhang[3], Changhong Yang[4], Fei Yin[1], Yue Ma[1,*], Tiejun Shui[5,*]

***: correspondence:**
Email: gordonrozen@scu.edu.cn (Yue Ma), shuitiejunynjkedu@163.com (Tiejun Shui)

Supplementary Table S1. Air quality station in Sichuan Basin

Site code	City	Longitude	Latitude
1431A	Chengdu	103.9728	30.72358
1432A	Chengdu	104.176	30.6872
1433A	Chengdu	104.079	30.5706
1434A	Chengdu	104.1114	30.63
1435A	Chengdu	104.074	30.685
1436A	Chengdu	104.026	30.6544
1437A	Chengdu	104.054	30.6578
1438A	Chengdu	103.613	31.0283
1878A	Mianyang	104.7778	31.4747
1879A	Mianyang	104.7536	31.4539
1880A	Mianyang	104.6717	31.4656
1881A	Mianyang	104.7283	31.5072
1882A	Yibin	104.5969	28.8194
1883A	Yibin	104.6225	28.76611
1884A	Yibin	104.6061	28.7867
1885A	Yibin	104.6417	28.76389
1886A	Yibin	104.5761	28.71583
1887A	Yibin	104.6789	28.7989
1893A	Luzhou	105.4306	28.9583
1894A	Luzhou	105.4436	28.9026
1895A	Luzhou	105.4322	28.8558
1896A	Luzhou	105.4322	28.8833

1897A	Zigong	104.7547	29.3628
1898A	Zigong	104.7214	29.3586
1899A	Zigong	104.7747	29.3564
1900A	Zigong	104.7692	29.3411
1901A	Deyang	104.4219	31.1208
1902A	Deyang	104.3883	31.1333
1903A	Deyang	104.4053	31.1167
1904A	Deyang	104.3539	31.1108
1905A	Nanchong	106.056	30.8064
1906A	Nanchong	106.0789	30.8023
1907A	Nanchong	106.1031	30.8217
1908A	Nanchong	106.1064	30.7856
1909A	Nanchong	106.0642	30.7636
1910A	Nanchong	106.1087	30.8388
2523A	Guangyuan	105.8945	32.4535
2524A	Guangyuan	105.8242	32.4429
2525A	Guangyuan	105.8153	32.4246
2526A	Guangyuan	105.8624	32.4285
2527A	Suining	105.7519	30.5797
2528A	Suining	105.68	30.6133
2529A	Suining	105.8161	30.6347
2530A	Suining	105.5956	30.475
2531A	Neijiang	105.0331	29.5953
2532A	Neijiang	105.0717	29.5947
2533A	Neijiang	105.0653	29.5817
2534A	Neijiang	105.0406	29.5822
2535A	Leshan	103.7705	29.5467
2536A	Leshan	103.7627	29.5844
2537A	Leshan	103.7506	29.6007
2538A	Leshan	103.757	29.5634
2539A	Meishan	103.8986	30.0506
2540A	Meishan	103.8416	30.0874
2541A	Meishan	103.8495	30.0691
2542A	Meishan	103.8341	30.062
2543A	Guang’an	106.631	30.518
2544A	Guang’an	106.6303	30.4576
2545A	Guang’an	106.6271	30.4663
2546A	Guang’an	106.6406	30.4844
2547A	Guang’an	106.6388	30.4551
2548A	Dazhou	107.5272	31.2797
2549A	Dazhou	107.525	31.215
2550A	Dazhou	107.4967	31.2108
2551A	Dazhou	107.5069	31.1956
2552A	Dazhou	107.4611	31.2058

2553A	Ya’an	103.009	30.0125
2554A	Ya’an	103.0013	29.9899
2555A	Ya’an	103.0109	29.9834
2556A	Ya’an	103.0001	29.9816
2557A	Bazhong	106.7583	31.8475
2558A	Bazhong	106.7389	31.8711
2559A	Bazhong	106.7594	31.8531
2560A	Bazhong	106.7608	31.8456
2561A	Ziyang	104.6617	30.1366
2562A	Ziyang	104.6356	30.1506
2563A	Ziyang	104.6289	30.1377
2564A	Ziyang	104.6469	30.1142
2565A	Ziyang	104.6294	30.1259
2880A	Chengdu	104.0239	30.65639
2902A	Guang’an	106.6351	30.4865
2914A	Bazhong	106.7514	31.87889
2915A	Bazhong	106.7619	31.85806
3027A	Zigong	104.7228	29.33972
3028A	Zigong	104.7778	29.36028
3136A	Chengdu	104.2725	30.5589

Supplementary Table S2. Meteorological monitoring station of Sichuan Province

Code	Latitude	Longitude
56038	32.98333	98.1
56079	33.58333	102.9667
56144	31.8	98.58333
56146	31.61667	100
56152	32.28333	100.3333
56167	30.98333	101.1167
56172	31.9	102.2333
56173	32.8	102.55
56178	31	102.35
56182	32.66667	103.6
56187	30.75	103.8667
56188	31	103.6667
56196	31.45	104.7333
56247	30	99.1
56251	30.93333	100.3167
56257	30	100.2667
56287	29.98333	103
56357	29.05	100.3
56374	30.05	101.9667
56385	29.51667	103.3333
56386	29.56667	103.75
56459	27.93333	101.2667
56462	29	101.5
56475	28.65	102.5167
56479	28	102.85
56485	28.26667	103.5833
56492	28.8	104.6
56565	27.43333	101.4833
56571	27.9	102.2667
56666	26.56667	101.7167
56671	26.65	102.25
57206	32.41667	105.9
57237	32.06667	108.0333
57306	31.58333	105.9833
57313	31.86667	106.7667
57328	31.2	107.5
57405	30.5	105.55
57411	30.73333	106.1167
57503	29.61667	105.1167
57604	28.78333	105.3833
57608	28.16667	105.4333

Supplementary Table S3. City-level meteorological conditions of 17 cities in the Sichuan Basin (daily data calculating by arithmetic mean)

city	Temperature	Relative humidity	Sunshine	Wind	Rainfall
Chengdu	16.84	81.39	2.99	1.31	2.59
Guangyuan	16.65	70.48	3.73	1.68	2.45
Mianyang	15.88	69.80	3.66	1.85	2.20
Deyang	17.62	75.57	3.21	1.56	2.34
Nanchong	18.26	75.12	3.67	1.48	2.75
Guang'an	18.50	78.73	3.55	1.53	3.26
Suining	18.18	79.33	3.24	1.41	2.53
Neijiang	18.61	80.22	3.12	1.33	2.54
Leshan	11.93	81.10	3.27	1.95	3.60
Zigong	18.93	79.10	3.05	1.23	2.71
Luzhou	18.53	85.90	3.34	1.70	3.50
Yibin	18.47	78.92	2.96	1.13	3.04
Bazhong	17.26	73.47	4.37	1.39	3.01
Dazhou	17.94	75.71	3.58	1.26	3.40
Ziyang	18.51	80.16	3.13	1.35	2.47
Meishan	17.00	78.79	2.88	1.31	3.20
Ya'an	13.24	77.79	3.19	1.75	3.69

Supplementary Table S4. Over-dispersion test for the HFMD cases time series of each city

City	Statistics	*P*-value
Chengdu	30459.75	*P*<0.05
Zigong	2812.00	*P*<0.05
Luzhou	3744.59	*P*<0.05
Deyang	5963.46	*P*<0.05
Mianyang	5286.25	*P*<0.05
Guangyuan	3914.17	*P*<0.05
Suining	3371.40	*P*<0.05
Neijiang	3334.90	*P*<0.05
Leshan	3811.06	*P*<0.05
Nanchong	5970.70	*P*<0.05
Meishan	7061.87	*P*<0.05
Yibin	2870.14	*P*<0.05
Guang'an	4884.38	*P*<0.05
Dazhou	5433.44	*P*<0.05
Ya'an	4694.80	*P*<0.05
Bazhong	6453.01	*P*<0.05
Ziyang	3880.49	*P*<0.05

Supplementary Text S1. Sensitivity Analysis (Take O_3 as example)

Combined with prior knowledge, this study adopted a systematic analysis strategy to determine the parameter settings of the DLNM model. The indexes of parameter evaluation were the average of estimates (AOE) and the sum of the quasi-Akaike information criterion (QAIC). AOE was used to measure whether the selection of parameters caught a systematic bias in estimates. The sum of QAIC (QAICs) was used to measure whether the goodness of fit of the model has improved. Based on the results of AOE and QAICs, we determined the parameters of DLNM by comprehensively considering the simplicity and rationality of the model.

1. The choice of the degrees of freedom (*df*) controlling the long-term trends and seasonality

We selected natural cubic spline (*ns*) to control the long-term trends and seasonality. The degrees of freedom (*df*) was changed from 1 to 10 per year. Figure S1 showed that when the degrees of freedom exceeded 8*df*/year, the QAICs and AOE became stable. So, we chose 8*df*/year of the natural spline to control the long-term trends and seasonality.

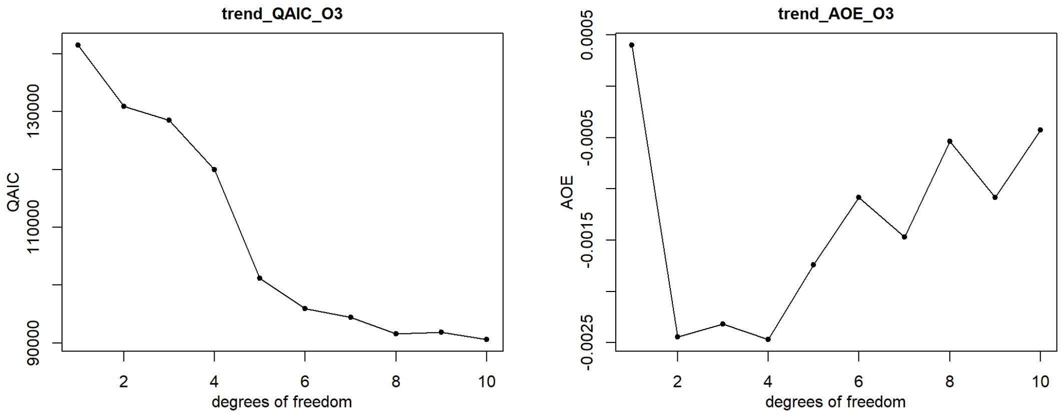

Supplementary Figure S1. The QAICs (left panel) and AOE (right panel) of the fitted model by changing the degrees of freedom of natural spline

2. The choice of the form of confounders

After univariate analysis, the sunshine duration, rainfall, SO_2, and NO_2 were selected as confounders. With no confounding variables included as the reference model, we set up eight models for each confounding variable (Table S5). Combining the results of all variables, we selected C2 for the sunshine duration, SO_2, and NO_2. C7 for rainfall. Thus, sunshine duration, SO_2, and NO_2 were set by calculating a simple moving average with 0-14 lag days. Rainfall was set by calculating the exponential moving average with a natural spline of 3 *df* with 0-14 lag days.

Supplementary Table S5. The parameter settings of confounder

Notation	association	Lag days	Constraint forms of lag days
C0	-	-	-
C1	Linear	4	-
C2	Linear	4-10	SMA
C3	Linear	4-10	EMA
C4	Linear	4-10	*ns* with 4 df
C5	Nonlinear	4	-
C6	Nonlinear	4-10	SMA
C7	Nonlinear	4-10	EMA
C8	Nonlinear	4-10	*ns* with 4 df

Note: *ns*: natural cubic splines SMA: simple moving average EMA: exponential moving average.

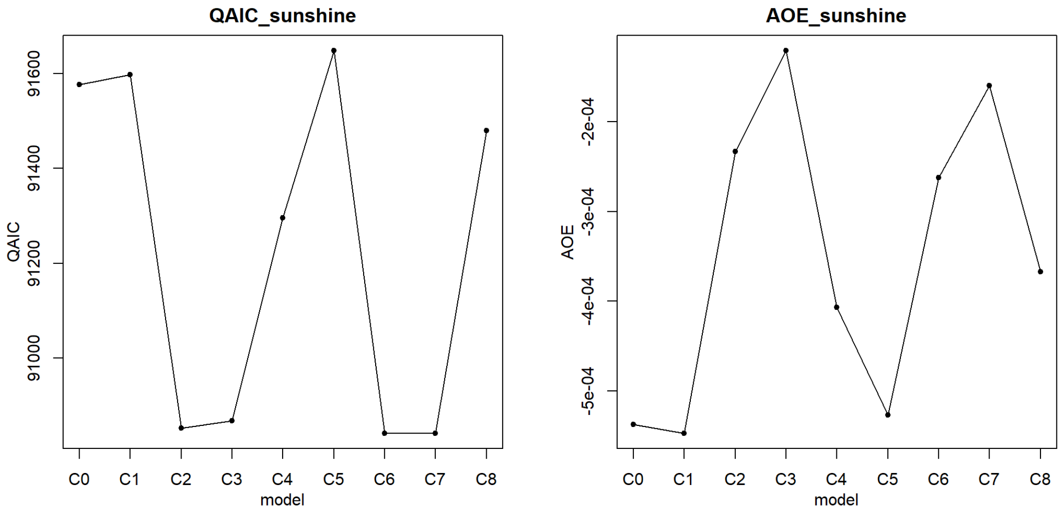

Supplementary Figure S2. The QAICs (left panel) and AOE (right panel) of different fitted model settings of sunshine duration (O_3 as example)

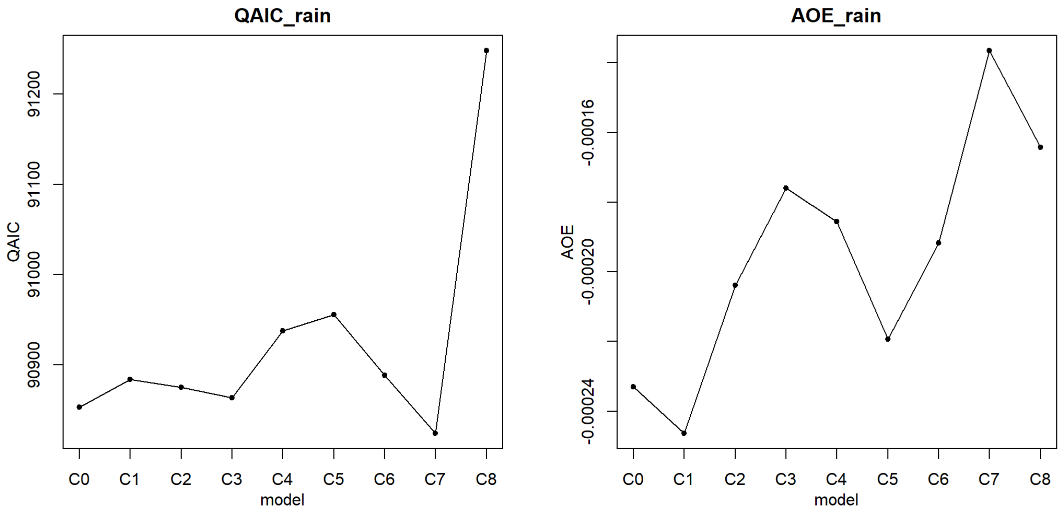

Supplementary Figure S3. The QAICs (left panel) and AOE (right panel) of different fitted model settings of rainfall (O_3 as example)

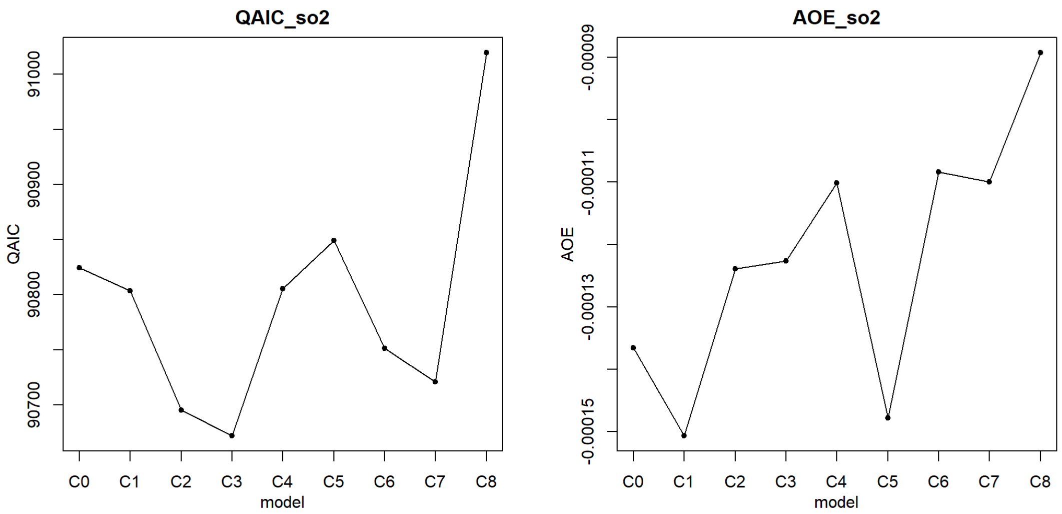

Supplementary Figure S4. The QAICs (left panel) and AOE (right panel) of different fitted model settings of SO_2 (O_3 as example)

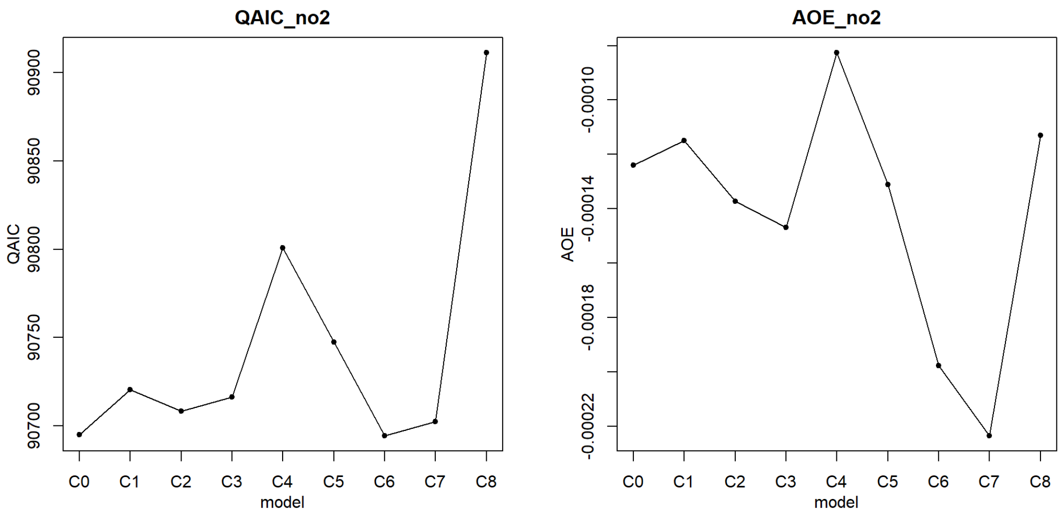

Supplementary Figure S5. The QAICs (left panel) and AOE (right panel) of different fitted model settings of NO_2 (O_3 as example)

3. The choice of the form of the autoregressive term

For the setting of autoregressive terms, we considered different lag orders and inclusion forms. Finally, with no autoregressive term included as the reference model, we set up six models (Table S6). According to the lowest QAICs, we selected A2 for the autoregressive term.

Supplementary Table S6. The parameter settings of autoregressive term

Notation	Lag orders	Scale	Inclusion forms
A0	-	-	-
A1	Lag 1~2	Original scale	None
A2	Lag 1~2	Logarithmic scale	None
A3	Lag 1~2	Logarithmic scale	SMA
A4	Lag 4~10	Original scale	None
A5	Lag 4~10	Logarithmic scale	natural cubic splines with 4 *df*
A6	Lag 4~10	Logarithmic scale	SMA

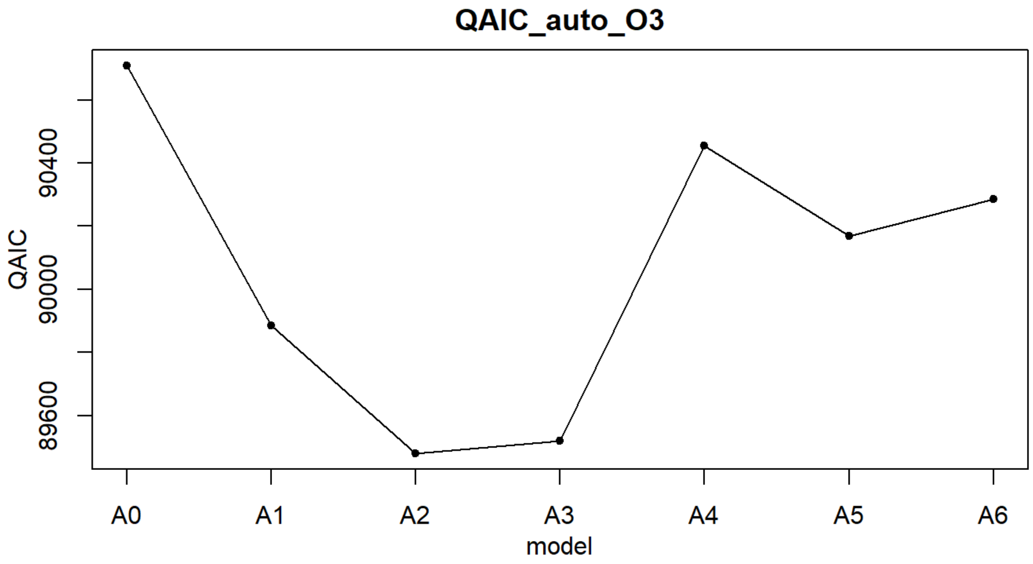

Supplementary Figure S6. The QAICs of different fitted model settings of autoregressive term

4. The choice of the lag structure of O_3 on HFMD

To comprehensively explore the structure of lag effects, we adopted unrestricted lag distribution models for 17 cities of the Sichuan Basin, and systematically conducted the corresponding effect estimates of O_3 with lag days from 0 to 30 days. Based on the AOE in Figure S7 and the incubation period of HFMD, we selected 0 to 14 days as the lag period of this study. In addition, the distribution of the lag-response relationships was set as a natural cubic spline function, with 3 to 5 degrees of freedom (*df*). In the end, we chose *ns* with 3 *df* for the lag distribution for all variables.

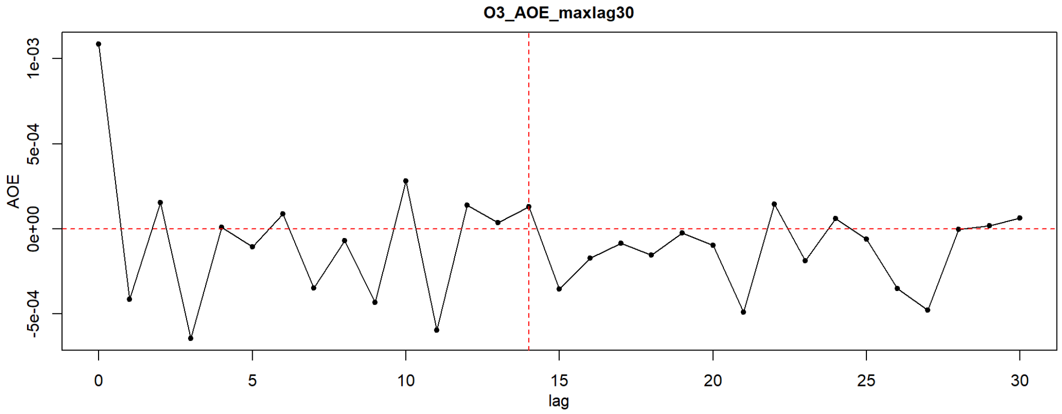

Supplementary Figure S7. The AOE of different lag days (0-30 days) of the fitted model

Supplementary Table S7. The QAICs of different *df* of natural cubic spline

df	**QAICs**
3	89061.49
4	89079.35
5	89095.96

5. The choice of equal knots of the exposure-response relationship of O_3 on HFMD

The exposure-response relationships were commonly constrained with a natural cubic spline. So, we performed the sensitivity analysis by setting up the exposure-response relationship with natural cubic spline functions, 2-4 equal knots, and calculating the QAICs (Table S8.). Considering all the results of the variables, we finally selected the spline function with two equal knots.

Supplementary Table S8. The QAICs of different number of equal knots of natural cubic spline

The number of equal knots	**QAICs**
2	89062.01
3	89106.26
4	89149.89

Supplementary Table S9. The Multivariate meta-regression model results of temperature on HFMD

		LR test			Cochran Q test			model fit		Heterogeneity	
Exposure	Modifier	$stats$	df	P	Q	df	P	AIC	ΔAIC	I^2	ΔI^2
**Single meta-predictor model**											
Temperature	reference	-	-	-	69.85	48	0.021	134.89	-	31.3	-
	temperature	8.64	3	0.034	58.89	45	0.080	132.25	-2.64	23.6	-7.7
	relative humidity	6.31	3	0.098	61.73	45	0.049	134.58	-0.31	27.1	-4.2
	sunshine duration	4.53	3	0.210	63.00	45	0.039	136.36	1.47	28.6	-2.7
	wind speed	4.03	3	0.258	65.70	45	0.024	136.85	1.96	31.5	0.2
	rainfall	0.23	3	0.972	69.74	45	0.010	140.65	5.76	35.5	4.2
**Multiple meta-predictors model**											
Temperature	Temperature+ Relative humidity	14.85	6	0.021	51.02	42	0.16	132.03	-2.86	17.7	-13.6

Supplementary Table S10. The Multivariate meta-regression model results of wind speed on HFMD

		LR test			Cochran Q test			model fit		Heterogeneity	
Exposure	Modifier	*stats*	*df*	*P*	*Q*	*df*	*P*	*AIC*	ΔAIC	I^2	ΔI^2
**Single meta-predictor model**											
Wind speed	reference	-	-	-	73.73	48	0.010	149.23	-	34.9	-
	temperature	3.52	3	0.318	65.07	45	0.027	151.71	2.48	30.8	-4.1
	relative humidity	9.19	3	0.027	53.94	45	0.170	146.04	-3.19	16.6	-18.3
	sunshine duration	3.24	3	0.355	64.42	45	0.030	151.98	2.75	30.1	-4.8
	wind speed	0.77	3	0.857	73.17	45	0.005	154.46	5.23	38.5	3.6
	rainfall	2.57	3	0.462	71.02	45	0.008	152.65	3.42	36.6	1.7
**Multiple meta-predictors model**											
Wind speed	-	-	-	-	-	-	-	-	-	-	-

Supplementary Table S11. The Multivariate meta-regression model results of relative humidity on HFMD

		LR test			Cochran Q test			model fit		Heterogeneity	
Exposure	Modifier	$stats$	df	P	Q	df	P	AIC	ΔAIC	I^2	ΔI^2
**Single meta-predictor model**											
Relative humidity	reference	-	-	-	104.59	48	<0.001	149.92	-	54.1	0
	temperature	2.87	3	0.412	98.75	45	<0.001	153.05	3.13	54.4	0.3
	relative humidity	1.39	3	0.708	101.07	45	<0.001	154.53	4.61	55.5	1.4
	sunshine duration	4.97	3	0.174	95.49	45	<0.001	150.95	1.03	52.9	-1.2
	wind speed	3.02	3	0.388	100.53	45	<0.001	152.90	2.98	55.2	1.1
	rainfall	2.04	3	0.564	96.97	45	<0.001	153.88	3.96	53.6	-0.5
**Multiple meta-predictors model**											
Relative humidity	-	-	-	-	-	-	-	-	-	-	-

Supplementary Table S12. The Multivariate meta-regression model results of $PM_{2.5}$ on HFMD

		LR test			Cochran Q test			model fit		Heterogeneity	
Exposure	Modifier	$stats$	df	P	Q	df	P	AIC	ΔAIC	I^2	ΔI^2
**Single meta-predictor model**											
$PM_{2.5}$	reference	-	-	-	93.24	48	<0.001	156.60	-	48.5	-
	temperature	8.49	3	0.037	85.79	45	<0.001	154.11	-2.49	47.5	-1
	relative humidity	0.48	3	0.922	92.39	45	<0.001	162.12	5.52	51.3	2.8
	sunshine duration	3.98	3	0.264	87.16	45	<0.001	158.62	2.02	48.4	-0.1
	wind speed	3.37	3	0.338	88.77	45	<0.001	159.23	2.63	49.3	0.8
	rainfall	4.61	3	0.203	83.09	45	<0.001	158.00	1.40	45.8	-2.7
**Multiple meta-predictors model**											
$PM_{2.5}$	-	-	-	-	-	-	-	-	-	-	-

Supplementary Table S13. The Multivariate meta-regression model results of CO on HFMD

		LR test			Cochran Q test			model fit		Heterogeneity	
Exposure	Modifier	$stats$	df	P	Q	df	P	AIC	ΔAIC	I^2	ΔI^2
**Single meta-predictor model**											
CO	reference	-	-	-	102.27	48	<0.001	157.84	-	53.1	0
	temperature	6.07	3	0.108	98.11	45	<0.001	157.77	-0.07	54.1	1
	relative humidity	4.14	3	0.247	95.57	45	<0.001	159.70	1.86	52.9	-0.2
	sunshine duration	1.34	3	0.719	96.64	45	<0.001	162.50	4.66	53.4	0.3
	wind speed	4.76	3	0.190	95.18	45	<0.001	159.08	1.24	52.7	-0.4
	rainfall	3.06	3	0.382	98.79	45	<0.001	160.78	2.94	54.5	1.4
**Multiple meta-predictors model**											
CO	-	-	-	-	-	-	-	-	-	-	-

Supplementary Table S14. The Multivariate meta-regression model results of O_3 on HFMD

		LR test			Cochran Q test			model fit		Heterogeneity	
Exposure	Modifier	$stats$	df	P	Q	df	P	AIC	ΔAIC	I^2	ΔI^2
**Single meta-predictor model**											
O_3	reference	-	-	-	123.35	48	<0.001	150.48	-	61.1	-
	temperature	5.32	3	0.150	114.51	45	<0.001	151.17	0.69	60.7	-0.4
	relative humidity	7.97	3	0.047	108.55	45	<0.001	148.52	-1.96	58.5	-2.6
	sunshine duration	4.11	3	0.250	111.40	45	<0.001	152.38	1.90	59.6	-1.5
	wind speed	7.79	3	0.051	106.48	45	<0.001	148.70	-1.78	57.7	-3.4
	rainfall	9.09	3	0.028	106.09	45	<0.001	147.39	-3.09	57.6	-3.5
**Multiple meta-predictors model**											
O_3	Relative humidity+ Wind speed +Rainfall	23.26	9	0.006	77.79	39	<0.001	145.23	-5.25	49.9	-11.2
